# Delicate balance among thermal stability, binding affinity, and conformational space explored by single-domain V_H_H antibodies

**DOI:** 10.1038/s41598-021-98977-8

**Published:** 2021-10-18

**Authors:** Emina Ikeuchi, Daisuke Kuroda, Makoto Nakakido, Akikazu Murakami, Kouhei Tsumoto

**Affiliations:** 1 Department of Bioengineering, School of Engineering, The University of Tokyo, Tokyo, 108-8639 Japan; 2 Panasonic Corporation Technology Division, Kyoto, 619-0237 Japan; 3 Medical Device Development and Regulation Research Center, School of Engineering, The University of Tokyo, Tokyo, 108-8639 Japan; 4 Department of Chemistry and Biotechnology, School of Engineering, The University of Tokyo, Tokyo, Japan; 5 Department of Parasitology and Immunopathoetiology, Graduate School of Medicine, University of the Ryukyus, Okinawa, 903-0215 Japan; 6 Laboratory of Medical Proteomics, The Institute of Medical Science, The University of Tokyo, Tokyo, 108-8639 Japan

**Keywords:** Biophysical chemistry, Proteins, Molecular biophysics, Protein design

## Abstract

The high binding affinities and specificities of antibodies have led to their use as drugs and biosensors. Single-domain V_H_H antibodies exhibit high specificity and affinity but have higher stability and solubility than conventional antibodies as they are single-domain proteins. In this work, based on physicochemical measurements and molecular dynamics (MD) simulations, we have gained insight that will facilitate rational design of single-chain V_H_H antibodies. We first assessed two homologous V_H_H antibodies by differential scanning calorimetry (DSC); one had a high (64.8 °C) and the other a low (58.6 °C) melting temperature. We then generated a series of the variants of the low stability antibody and analyzed their thermal stabilities by DSC and characterized their structures through MD simulations. We found that a single mutation that resulted in 8.2 °C improvement in melting temperature resulted in binding affinity an order of magnitude lower than the parent antibody, likely due to a shift of conformational space explored by the single-chain V_H_H antibody. These results suggest that the delicate balance among conformational stability, binding capability, and conformational space explored by antibodies must be considered in design of fully functional single-chain V_H_H antibodies.

## Introduction

Antibodies are important molecules in our bodies, as they recognize foreign pathogens or antigens in a course of immune responses. The high binding affinities and specificities of antibodies also enable their use as drug candidates and biosensors. The sequences and structural features of antibodies vary depending on species^1–3^. For example, some antibodies from camelids have both heavy and light chain variable domains, as do conventional antibodies from humans, but camelids also have antibodies that lack the light chains that are termed single-domain V_H_H antibodies. Single-domain V_H_H antibodies combine the advantages of the specificity and affinity of conventional antibodies with high stability and solubility originating from nature of single-domain proteins^4,5^.

The antigen binding sites of conventional antibodies consist of six complementarity determining regions (CDRs) L1, L2, L3, H1, H2, and H3. The CDRs other than H3 adopt structures that are classified into limited conformations called canonical structures, and some residues in framework regions support these limited conformations^6–12^. Therefore, binding affinities can be affected by engineering not only residues in CDRs, but also residues in framework regions, as demonstrated in earlier work on antibody humanization^13^. CDR-H3 is located in the center of the antigen binding site, is the most diverse both in sequence and structure, and is the most critical of the CDRs to antigen recognition^14–17^. In contrast, the antigen binding site of single-domain V_H_H antibodies consists of only three CDRs. Thus, the framework regions of single-domain V_H_H antibodies are sometimes directly involved in recognition of antigens^18–21^.

Computational methods as well as in vitro library technologies are now frequently used to engineer antibodies^22–31^. For instance, Kiyoshi et al. computationally predicted affinity-enhancing mutations and experimentally demonstrated that some of the predicted mutations indeed improved the binding affinity of the antibody^32^. Olson et al. compared sequence fitness profiles, generated by computational design calculations and experimental mutagenesis, of a single-domain antibody that exhibited an unusually high melting temperature (T_m_ = 85.0 °C), demonstrating accuracies and limitations of current computational models^33^. Previous studies also demonstrated that single-domain antibodies can be engineered to improve the thermal and colloidal stability^4,34,35^. In one such example, based on molecular dynamics (MD) simulations, Bekker et al. showed that the fraction of native contacts, or Q-value, that had been employed in studies of protein folding^36^ could be used as an evaluation metric to identify residues important for thermal stability of single-domain antibodies^37^. In another study, Zabetakis et al. demonstrated that the stability-enhancing mutations identified by Bekker et al. led to reductions of the binding affinities to antigen^38^. This demonstrated the difficulty of simultaneously improving thermal stability and binding affinity and showed that our understanding of the relationships between binding capability and other physical properties is not yet sufficient to design antibodies in a rational manner.

In this study, we employed physicochemical measurements, structural modeling and MD simulations to analyze two homologous, single domain V_H_H antibodies that differ in melting temperatures by 6.2 °C. We sought to determine what underlies different thermal stabilities of these two antibodies that differ at a limited set of residues, to understand the tradeoff between thermal stability and function, and to identify the features that result in both higher affinity and higher thermal stability. Our results showed that there is a delicate balance among thermal stability, binding affinity, and explored conformational space that must be considered in engineering of single-domain V_H_H antibodies.

## Results

### Single-domain V_H_H antibodies to serum albumin with nine amino acid differences have dramatically different thermal stabilities and binding affinities

**Figure 1:**
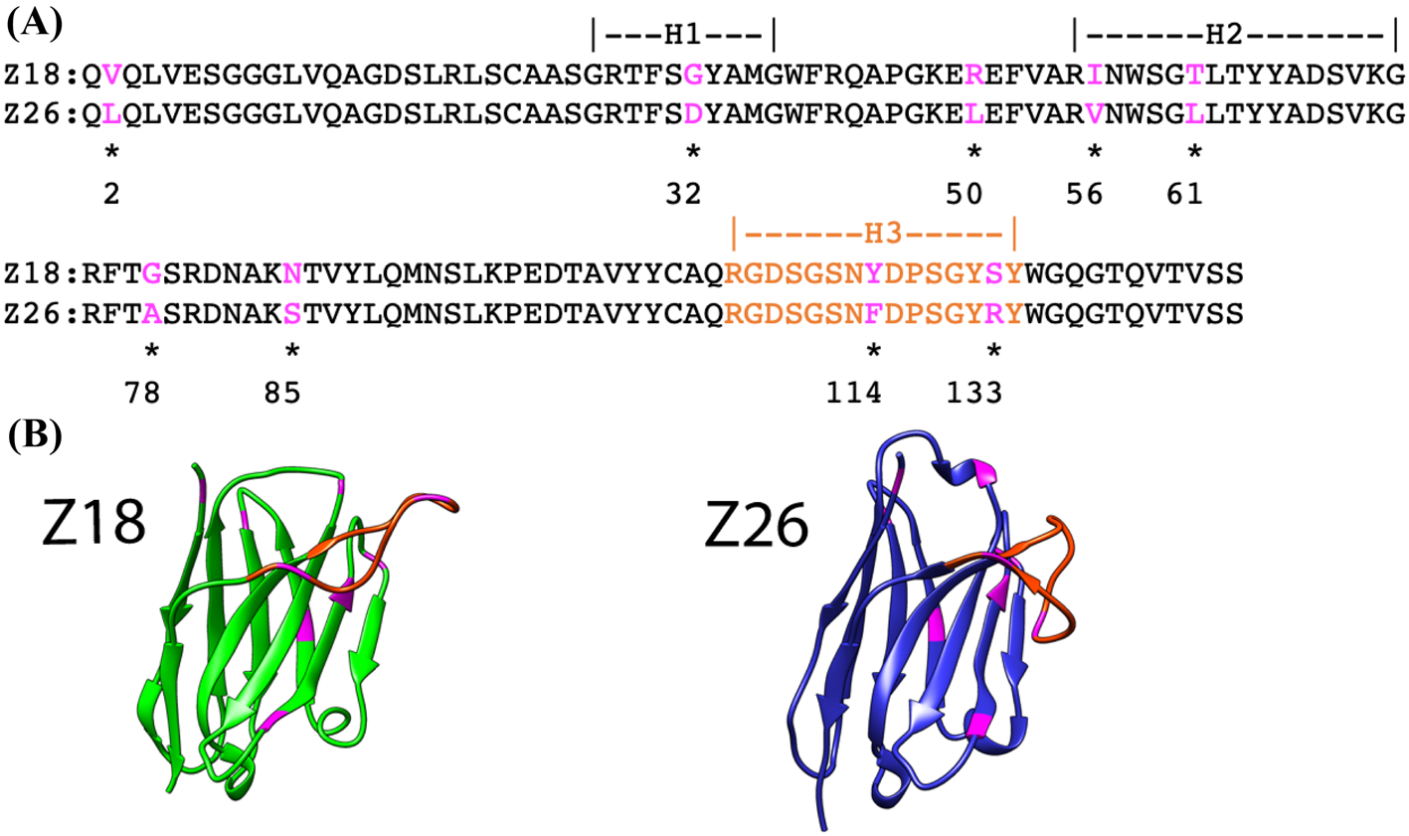
Comparison of amino acid sequences and predicted structures of the single-domain V_H_H antibodies Z18 and Z26. (**A**) Amino acid sequences of Z18 and Z26. CDR regions are indicated and amino acids that are not identical are indicated by asterisks. Residues are numbered according to the IMGT numbering scheme^39^. (**B**) Homology models of Z18 and Z26. The CDR-H3 and mutational positions are shown in orange and magenta, respectively. Protein images were generated with UCSF Chimera^40^.

This study focused on two single-domain V_H_H antibodies, Z18 and Z26, that were selected in-house for affinity to human serum albumin (HSA). Pair-wise sequence comparison revealed that Z18 and Z26 are highly homologous; only nine amino acids are different between the two antibodies (Fig. 1A). Computational structure predictions based on each sequence revealed highly homologous framework structures with distinct conformations of the CDR-H3 (Fig. 1B).

An analysis by differential scanning calorimetry (DSC) showed that Z18 had lower thermostability than Z26. The difference in melting temperature (T_m_) was 6.2 °C (Table 1). Assessment of the antigen binding by surface plasmon resonance (SPR) showed that the affinity of Z18 for HSA was 3 orders of magnitude lower than that of Z26 (1.75 × 10^–7^ M and 5.40 × 10^–10^ M for Z18 and Z26, respectively; Table 1 and Figure S1).

**Table 1:** Thermal stabilities and binding affinities of Z18 mutants and Z26.

	T_m_ (°C)	*k*_on_ (1/M·S)	*k*_off_ (1/s)	*K*_D_ (M)
Z18	58.6 ± 0.1	7.74 × 10^5^	0.259	3.34 × 10^–7^
Z18-V2L	57.8 ± 0.4	7.54 × 10^5^	0.276	3.66 × 10^–7^
Z18-G32D	57.8 ± 0.4	6.96 × 10^5^	0.120	1.72 × 10^–7^
Z18-R50L	60.9 ± 0.4	6.89 × 10^5^	0.284	4.12 × 10^–7^
Z18-I56V	55.1 ± 0.4	7.41 × 10^5^	0.220	2.96 × 10^–7^
Z18-T61L	55.5 ± 0.7	4.33 × 10^6^	0.037	8.63 × 10^–9^
Z18-G78A	66.8 ± 0.3	1.55 × 10^5^	1.101	7.11 × 10^–6^
Z18-N85S	56.2 ± 0.9	6.92 × 10^5^	0.238	3.44 × 10^–7^
Z18-Y114F	57.8 ± 0.8	7.45 × 10^6^	0.096	1.29 × 10^–8^
Z18-S133R	59.2 ± 0.6	1.16 × 10^6^	0.078	6.72 × 10^–8^
Z18-R50L/G78A/S133R	70.9 ± 0.1	1.76 × 10^6^	0.785	4.47 × 10^–7^
Z18-G32D/R50L/G78A/S133R	70.6 ± 0.2	5.16 × 10^5^	0.412	7.97 × 10^–7^
Z26	64.8 ± 0.4	1.51 × 10^6^	8.16 × 10^–4^	5.40 × 10^–10^

**Figure 2:**
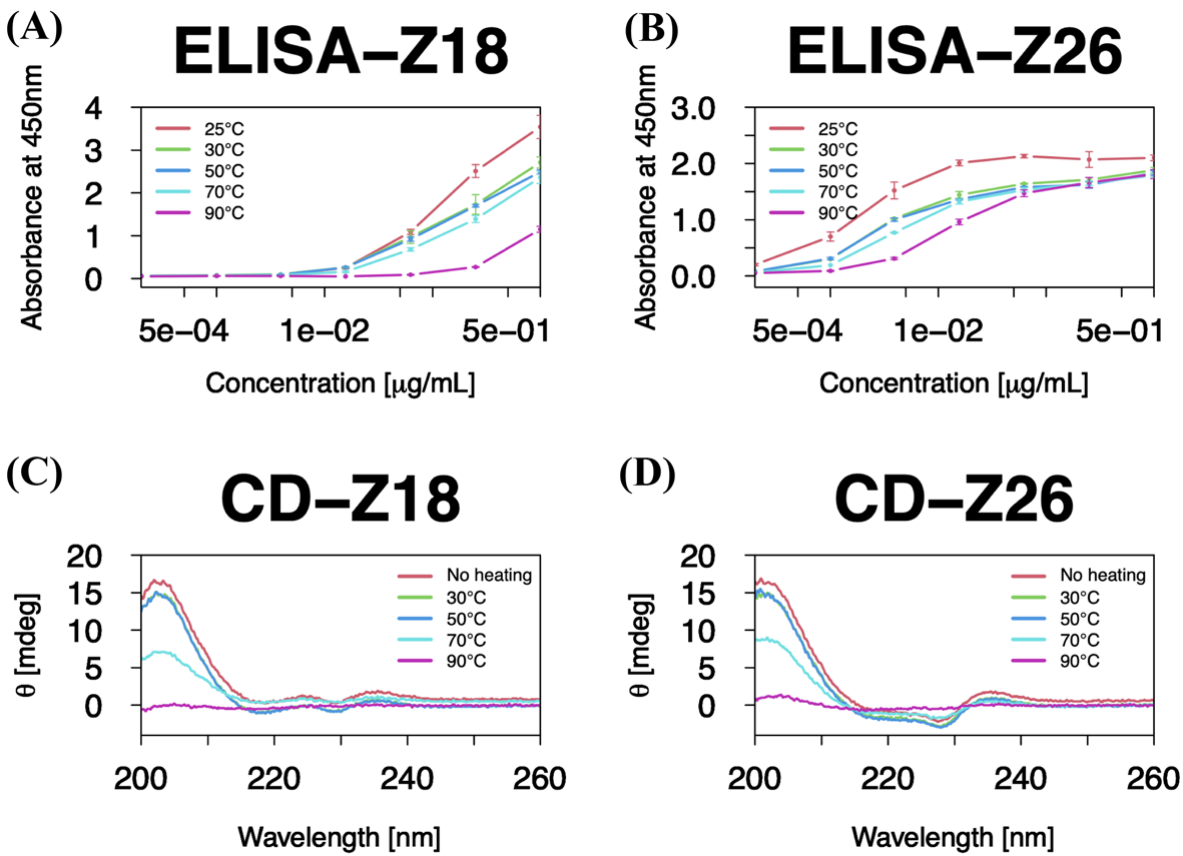
Binding capabilities and secondary structures of the single-domain V_H_H antibodies show similar trends between Z18 and Z26. (**A**,**B**) ELISA to quantify binding of (**A**) Z18 and (**B**) Z26 to HSA. ELISA was independently repeated three times, and the average values were plotted with the standard deviations. (**C**,**D**) CD spectra of (**C**) Z18 and (**D**) Z26. CD measurements were performed with 0.15 mg/mL of a sample in PBS. All graphs in this article were made by R packages^41^.

Unlike conventional antibodies, single-domain antibodies refold correctly after heat-induced denaturation^4^. Therefore, we used an enzyme-linked immunosorbent assay (ELISA) and circular dichroism (CD) to assess the binding abilities and conformational changes, respectively, after thermal stress at different temperatures (30 °C, 50 °C, 70 °C, 90 °C). Both antibodies showed similar trends in binding capabilities and CD spectra before and after the thermal stress (Fig. 2); both antibodies preserved the binding affinities and secondary structures up until 50 °C, whereas, when the temperature was elevated to 70 °C or 90 °C, the antibodies began to denature and lose their binding abilities. These results motivated us to investigate the origin of the difference in physicochemical properties between the two antibodies generated by the difference in very few amino acids.

### A single mutation in Z18 increases thermal stability but decreases binding affinity

To examine how the differences in sequence between the low stability Z18 and the high stability Z26 influence stability and function, we prepared nine mutants of Z18, each mutation altered a single residue to the amino acid observed in Z26. Thermal stabilities were measured by DSC (Table 1). The mutant G78A had the largest impact on the thermal stability; the T_m_ of this mutant was 8.2 °C higher than that of the wild-type Z18 and was 2.0 °C higher than that of wild-type Z26. When binding affinity was measured by SPR for these mutants, the most stable mutant Z18-G78A had considerably lower affinity than Z18 and Z26 (Table 1, Figures S1 and S2). Furthermore, the Z18-R50L mutant also improved the thermal stability by 2.3 °C while it had neutral effects on the binding affinity.

The Z18-T61L mutant was an order of magnitude tighter binder of HSA than Z18, but its T_m_ was lower than that of Z18 by 3.1 °C (Table 1), suggesting that, although single-domain V_H_H antibodies do not always use CDR-H2 to interact with the antigens, the T61L mutation, which is located in CDR-H2, may have a role in the antigen binding. Similarity, the Z18-Y114F and Z18-S133R mutants, which are located in CDR-H3, exhibited slightly better binding affinities than Z18, implying a role of these residues in the antigen recognition. Interestingly, the S133R mutation also slightly affect the thermal stability favorably by 0.6 °C. When the thermally stabilizing mutations (R50L/G78A/S133R) were combined, the T_m_ was higher than that of Z18-G78A by 4.1 °C, and the binding affinity became comparable to that of Z18.

A previous study demonstrated that a combination of 2 mutations, each of which showed decreased affinity or no expression, was able to improve the binding affinity of an antibody^42^. Notably, those 2 mutations were spatially far from each other. To explore such corporative effects of distantly located mutations, we focused on the G32D mutation, which is located in CDR-H1, but did not affect the binding affinity (Table 1). We then combined four mutations, G32D, R50L, G78A, and S133R, which resulted in improvement of the thermal stability by 12.0 °C, but the binding affinity still remained comparable to that of Z18.

Together, these data suggest that each mutation has a distinctive role in either thermal stability or binding affinity (Table 1 and Figure S1). In most cases, mutations in CDRs influenced binding affinity and those in framework regions altered thermal stability. Some mutations (e.g., V2L and G32D) were neutral. However, it is worth noting that we focused only on thermal stability and binding affinity in this study, but other factors, such as colloidal stability and specificity, could be affected by these mutations^43^.

### Conformational space of a CDR governs thermal stability and binding affinity

To gain molecular insights into roles of the stabilizing mutation G78A, we visually inspected the predicted structures of Z18 and Z26. The difference in chemical structure between the two amino acids is a single methyl group present in Ala but not in Gly. This methyl group appears to fill a cavity observed inside the predicted structure of Z18 (Figure S3). Core regions of protein structures are known to be well packed^44^, and even a small cavity can lead to destabilization. Filling such a cavity in the antibody structure could change its dynamics. Therefore, we further employed 1.1 μs MD simulations to investigate roles of mutations in conformational dynamics. Since epitope information of the antigen is not available, we limited ourselves to computational assessments of the unbound states of the wild-type antibodies and the mutants. Based on the experimental results, we computationally analyzed Z18, Z18-G78A, Z18-G32D/R50L/G78A/S133R, and Z26. We performed 5 independent simulations with different initial velocities for each antibody (total aggregated time ~ 22 μs).

**Figure 3:**
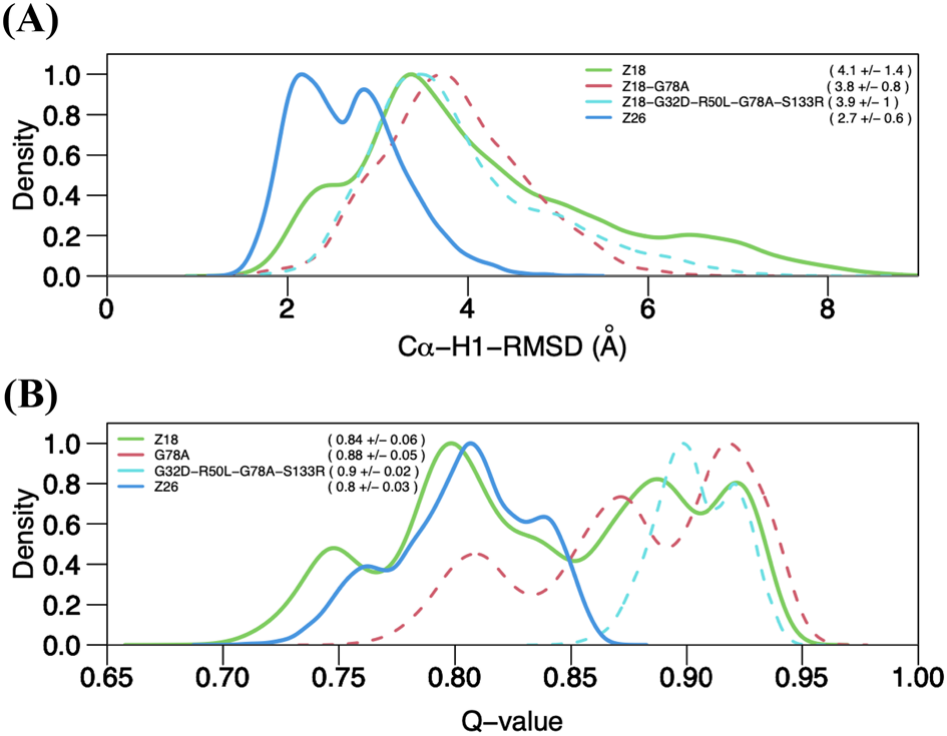
Dynamics of CDR-H1 of Z18 and Z26 differ. Kernel density estimation of (**A**) Cα-RMSDs of CDR-H1 and (**B**) Q-values computed based on the last 500 ns simulations for Z18, Z18-G78A, Z18-G32D/R50L/G78A/S133R, and Z26. The last 500 ns trajectories of each system were merged in each plot. Averages and standard deviations are indicated in parenthesis. Y-axis was normalized to a range between 0 and 1 for each system.

The root mean square deviations (RMSDs) of Cα atoms during the simulations were determined (Figure S4). All showed standard deviations less than 1.0 Å after 600 ns, suggesting the convergence of each trajectory (Table S2). When we split each trajectory after 600 ns in half, we observed substantial overlap between the first half and last half of each trajectory (Figure S5), supporting our claim that our simulations were converged well. However, Cα-RMSD of RUN-1 of Z18 deviated largely from those of the other 4 simulations (Figure S5). Therefore, in the analyses below, we did not consider the first 600 ns of the trajectories of each antibody and the entire trajectory of RUN-1 of Z18. Antibodies recognize antigens through their CDRs, and previous studies suggested that CDRs impact thermal stability^34,45^. Therefore, we also computed Cα-RMSDs of each CDR to further quantify the dynamics. We first superposed the Cα-atoms of the framework region and then computed Cα-RMSDs of each CDR. Of the CDRs, CDR-H3 exhibited the largest movements, followed by CDR-H1 and CDR-H2 (Figure S6). Interestingly, the RMSDs of the CDR-H1 of Z18 and Z26 clearly differed: Z18, which has the lower T_m_ had larger fluctuations than Z26 (Figure S6). This difference was more evident when conformational space was described by the kernel density estimation as frequency distributions of the RMSDs (Fig. 3A). Z18 as well as the stabilized mutants exhibited broader conformational space than Z26.

**Figure 4:**
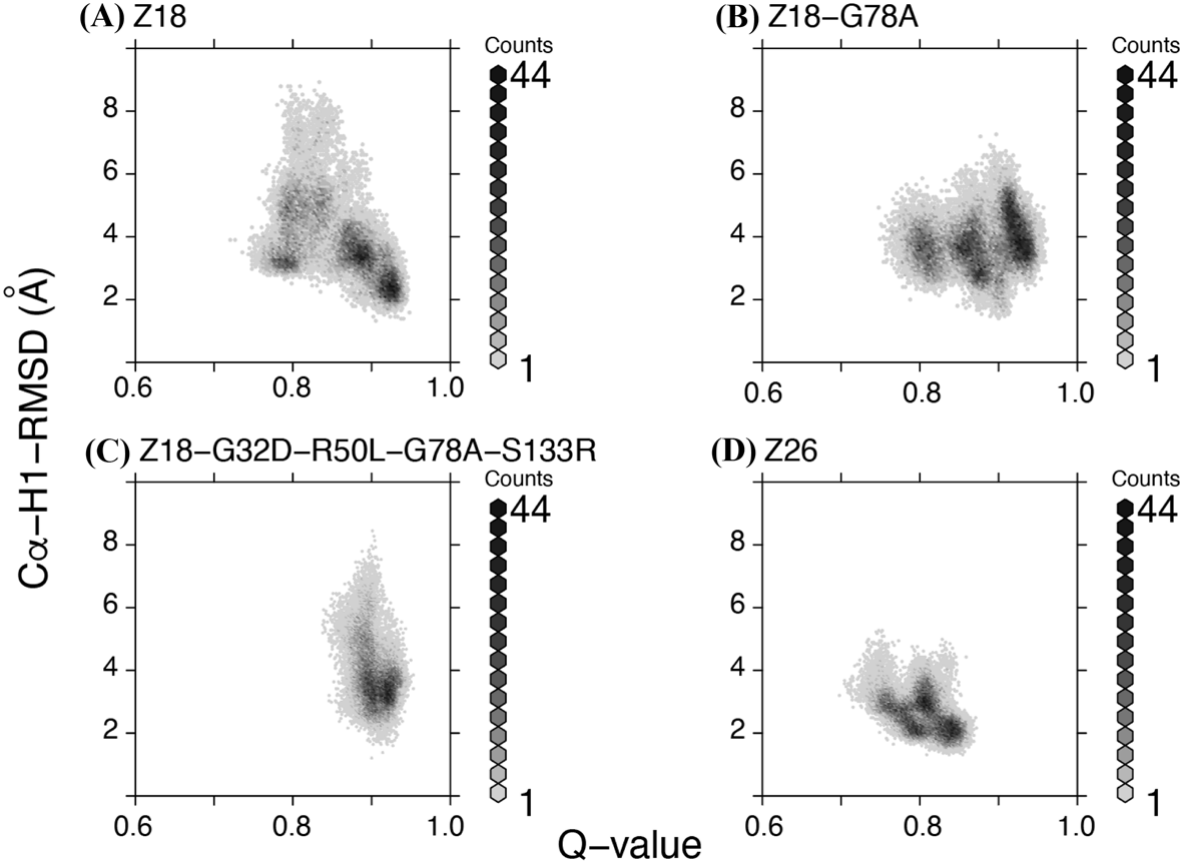
Conformational space explored correlates with thermal stability. Plots of Q-value versus Cα-RMSDs of CDR-H1 of indicated single-domain V_H_H antibodies. The last 500 ns trajectories of five simulations were merged in each plot.

A previous study suggested that fraction of native contacts, or Q-value, are correlated well with thermal stabilities of single-domain antibodies^37^. In the five simulations performed for each system, we observed variations of Q-value (Figure S7). Two trajectories of Z18, the antibody with the lowest stability, had higher Q-values on average than the averages of the Z26 trajectories. On the other hand, the most stable antibody, Z18-G32D/R50L/G78A/S133R, had consistently the higher Q-values (~ 0.9) throughout the simulations. When conformational space was described in terms of Q-values of all the 5 trajectories together for each antibody, Z18 exhibited broader distribution than Z26 (Fig. 3B). Notably, the stabilized mutants explored a higher range of conformational space than Z18 and Z26. These data suggest that although Q-value analysis at 298 K, conditions that approximate room temperature, is not an absolute method for assessing thermal stability, it could inform rational antibody design. Indeed, in plots of Q-value versus Cα-RMSD of CDR-H1, we observed strong correlations (Fig. 4).

Both Q-values and Cα-RMSDs of CDR-H1 indicate that the space explored by Z18 is larger than that explored by Z26 (Figs. 3, 4A,D). The single mutation Gly to Ala mutation at the position 78 of Z18 resulted in comparable stability to Z26 but binding affinity was sacrificed (Table 1). This observation may be rationalized by the fact that the G78A mutation shifted the conformational space explored by Z18 (Figs. 3 and 4). The Z18-G32D/R50L/G78A/S133R mutant, which was more thermodynamically stable than Z26 explored similar conformational space to Z18-G78A, with a narrower range of the Q-value (Figs. 3 and 4C). The binding affinity of the Z18-G32D/R50L/G78A/S133R mutant was tighter than that of Z18-G78A and comparable to that of Z18, but still inferior to that of Z26. Our simulations included only antibodies, and therefore the conformational spaces correspond to the conformational ensembles of unbound-state antibody structures. As the antigen binding could induce conformational changes in antibodies, understanding differences in binding affinities of Z18-G32D/R50L/G78A/S133R, Z18-G78A, and Z26 will require structural information of the antibody-antigen complexes.

## Discussion and conclusions

Two single-domain V_H_H antibodies that have high sequence homology, but different thermal stabilities, were analyzed through physicochemical measurements, structural modeling, and MD simulations. Our comparative analyses support the hypothesis that the thermal stability of antibodies can be increased by restricting conformational space. Previous works validated this approach by introducing disulfide bonds in antibody structures^46^. We demonstrated here that conformational restriction resulting from filling a cavity inside the structure of the Z18 antibody by mutation of Gly to Ala enhanced stability considerably.

Starting from the Z18 antibody, we were able to design more thermally stable antibodies, but these antibodies still had lower binding affinities for HSA than the Z26 antibody. Our MD simulations suggested that these antibodies Z18-G78A and Z18-G32D/R50L/G78A/S133R explored a higher range of conformational space, in terms of Q-value, than the higher affinity Z26 antibody, and still similar conformational space of CDR-H1 to that of Z18 rather than Z26. Thus, there seems to be a tradeoff between thermal stability and binding affinity, and conformational space explored by antibodies is a factor that governs the stability-function tradeoff.

**Figure 5:**
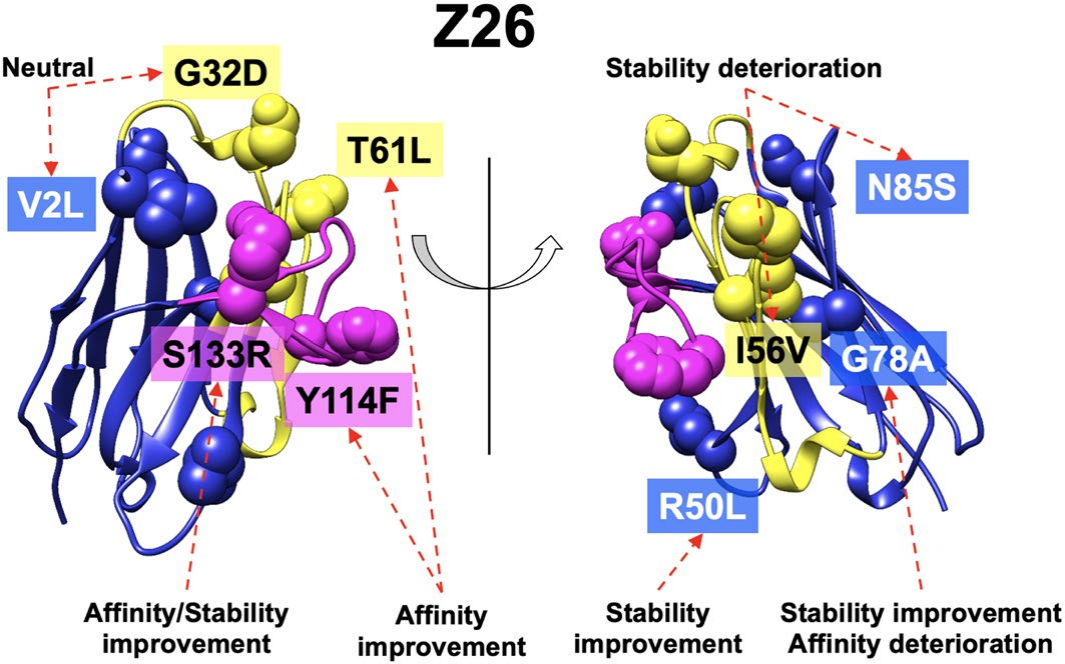
Correlations between the location and effect of the mutations. Residues that differ between Z18 and Z26 are shown as spheres. CDR-H1 and H2 are colored in yellow whereas CDR-H3 are in magenta.

Compared to Z18, Z26 had higher thermal stability and better binding affinity. Of the nine residues that differ between these two single-domain V_H_H antibodies, only a mutation in Z18 to the amino acid in Z26 (S133R) improved both of the thermal stability and the binding affinity. Mutations at two sites (R50L, G78A) had favorable effects on the thermal stability whereas mutations at two other sites (T61L, Y114F) contributed favorably to the binding affinity. Two mutations (I56V, N85S) decreased thermal stability with marginal effects on binding affinity. The other two mutations (V2L, G32D) were neutral. The locations and the effects of the mutations were summarized in Fig. 5. These results indicated that combination of mutations yields more than a simple additive effect.

Antibodies evolve in specific response to antigens through somatic hypermutation of their germline genes, resulting in the gradual accumulation of mutations in CDRs as well as in framework regions^47^. As demonstrated in this study, mutations in CDRs could direct affinity maturation whereas those in framework regions may improve physical stability. In this study, we further showed that mutations inside an antibody structure or in a framework region could modulate conformational space explored by antibodies. Previous studies showed that somatic mutations restrict conformational space^48–52^, which leads to better binding affinities by minimizing entropy loss upon antigen binding. On the other hand, a recent large-scale repertoire analysis suggest that somatic mutations do not typically result in rigidification^53^. In this context, we showed that rigidification of an antibody could stabilize an antibody, but instead scarify binding affinity, and there is a complex interplay between binding affinity and physical stability, in agreement with previous observations from the directed evolution of a single domain antibody^54,55^.

In summary, based on experimental measurements and computational techniques, we revealed that there is a delicate balance among thermal stability, binding affinity, and conformational space explored by single-domain V_H_H antibodies. Our results suggest that consideration of antibody structural dynamics is an important step in rational antibody design.

## Methods

### Expression and purification of antibodies

The DNAs encoding V_H_H antibodies Z18 and Z26 were incorporated into pRA2 vector^56^ with N-terminal pelB signal peptide and a C-terminal hexa-histidine tag. *E*. *coli* strain BL21 (DE3) cells (Novagen) were transformed with the expression vectors and grown at 28 °C in LB medium. Protein expression was induced by addition of 0.5 mM isopropyl β-d-1-thiogalactopyranoside when the optical density at 600 nm reached a value of 1.0. Cells were allowed to grow for an additional 16 h at 20 °C. The cells were harvested by centrifugation at 6500 × g for 10 min, and the cell pellet was resuspended in buffer A (20 mM Tris–HCl, 500 mM NaCl, pH 8.0) supplemented with 5 mM imidazole, after which cells were lysed with an ultrasonic disruptor (UD-201, TOMY) for 20 min. The cell lysate was centrifuged (40,000×*g* for 30 min) at 4 °C. The supernatant was filtered through an 0.8-μm pore-size filter and subsequently loaded onto a 1-mL Ni-NTA agarose column (Qiagen) equilibrated with buffer A. After washing with buffer A containing 20 mM imidazole, V_H_H antibodies were eluted from the column with buffer A supplemented with 300 mM imidazole. The eluate was subjected to size-exclusion chromatography using a HiLoad 26/600 Superdex 75 pg column (GE Healthcare) equilibrated with a buffer containing 20 mM TRIS–HCl, 200 mM NaCl.

### Antigen

The antigen, HSA, was purchased from Sigma-Aldrich Japan (Tokyo, Japan). The HSA was dissolved in a phosphate buffer and the HSA stock solution was prepared by extensive overnight dialysis at 4 °C.

### Surface plasmon resonance assays

The interactions between antibodies and HSA were analyzed by SPR on a Biacore T200 instrument (GE Healthcare). A research-grade CM5 Biacore sensor chip (GE Healthcare) was activated by a short treatment with *N*-hydroxysuccinimide/*N*-ethyl-*N*′-(3-dimethylaminopropyl) carbodiimide hydrochloride, followed by immobilization of antigen HSA at a surface density of approximately 1000 RU. The activated groups on the surface of the sensor were subsequently blocked by injecting 1 M ethanolamine as previously described^57^. Kinetic data were obtained by injecting increasing concentrations of antibodies over the sensor chip at a flow rate of 30 μl/min. The measurements were carried out in PBS containing 0.05% (v/v) Tween-20. Contact time and dissociation time were 4 min and 10 min, respectively. Data analysis was performed with the BIAevaluation software (GE Healthcare). Association (*k*_on_) and dissociation (*k*_off_) constants were calculated by a global fitting analysis assuming a Langmuir binding model and a stoichiometry of (1:1).

### Enzyme-linked immunosorbent assay

Microtiter plate wells were coated with HSA at 10 μg/ml overnight at 4 °C. The wells were washed and blocked with 3% skim milk/PBS supplemented with 0.05% Tween-20 (PBS-T) at room temperature for 1 h. Following three washes with PBS-T, V_H_H samples were added into wells and incubated at room temperature for 1 h. The wells were washed three times with PBS-T and bound antibody was detected with anti-His-tag mAb-HRP-DirecT (MBL) after incubation at room temperature for 1 h. The wells were washed three times with PBS-T and developed with TMB substrate mixture (Cosmobio). The reaction was stopped with Stop buffer (Cosmobio) after 20 min and the absorbance at 450 nm was measured.

### Circular dichroism spectra

CD spectra were recorded on a model J-820 CD spectrometer (JASCO). Far-UV CD measurements were performed with 0.15 mg/mL of a sample in PBS using a 1-mm cell and a bandwidth of 1 nm. Spectra were recorded five times for each sample.

### Differential scanning calorimetry

DSC measurements of samples prepared in PBS were carried out on a MicroCal PEAQ-DSC (Malvern) at a heating rate of 60 °C per h. To evaluate thermal stability, the temperature where heat capacity was maximum was determined as apparent T_m_ using the software MicroCal PEAQ-DSC software (Malvern).

### Modeling of V_H_H antibody structures

To model structures of the V_H_H antibodies, we divided the sequences into four parts (CDR-H1, CDR-H2, CDR-H3, and framework region) and used BLAST^58^ to search for each structural template in the RosettaAntibody database^59,60^, which was originally derived from the Protein Data Bank^61^. The sequences with the highest bit-scores in the database were chosen as templates (Table S1). These templates were brought together to generate a crude model of the single-domain antibodies. Next, we used the Relax optimization with harmonic constraints to minimize deviation from the template crystal structures as previously described^62^. The resulting V_H_H model structures were then used for the MD simulations to computationally assess the stabilities and dynamics of the antibodies.

### Molecular dynamics simulations

MD simulations of the four antibodies were performed using GROMACS 2019.4^63^ with the CHARMM36m force field^64^. The structures were solvated with TIP3P water^65^ in a rectangular box such that the minimum distance to the edge of the box was 15 Å under periodic boundary conditions. The protein charge was neutralized with added Na or Cl, and additional ions were added to imitate a salt solution of concentration 0.14 M. Each system was energy-minimized for 5000 steps with the steepest descent algorithm and equilibrated with position restraints of protein heavy atoms and NVT ensemble, where the temperature was increased from 50 to 298 K during 500 ps. Further non-restrained simulations were performed with the NPT ensemble at 298 K for 1.1 μs. The time step was set to 2 fs throughout the simulations. A cutoff distance of 12 Å was used for Coulomb and van der Waals interactions. Long-range electrostatic interactions were evaluated by means of the particle mesh Ewald method^66^. Covalent bonds involving hydrogen atoms were constrained by the LINCS algorithm^67^. A snapshot was saved every 100 ps. We repeated the same calculations 5 times with difference initial velocities.

Trajectory analysis was performed based on the last 500 ns trajectories. RMSDs were computed with the GROMACS package^63^. Q-value was computed with the MDTraj Python library^68^ using the equation below^36^:

$$ {\text{Q}}({\text{X}}) = \frac{1}{{\text{N}}}\mathop \sum \limits_{{({\text{i,j}}) \in {\text{S}}}} \frac{1}{{1 + \exp \left[ {\beta ({\text{r}}_{{{\text{ij}}}} ({\text{X}}) - \lambda {\text{r}}_{{{\text{ij}}}}^{0} )} \right]}} $$

where the sum runs over the *N* pairs of native contacts (*i,j*), *r*_*ij*_(*X*) is the distance between *i* and *j* in configuration *X*, ${\text{r}}_{{{\text{ij}}}}^{0}$ is the distance between *i* and *j* in the native state, β is a smoothing parameter taken to be 5 Å^−1^, and the factor λ accounts for fluctuations when the contact is formed, taken to be 1.8 for the all-atom simulations^36^.

## Supplementary Information

Supplementary Information.
