# Supplementary material for: Delicate balance among thermal stability, binding affinity, and conformational space explored by single-domain VHH antibodies
Source: Sci Rep. 2021 Oct 18;11:20624. doi: 10.1038/s41598-021-98977-8 (PMC8523659; doi:10.1038/s41598-021-98977-8)
Supplement: Supplementary file 1 — Supplementary Information. [file 41598_2021_98977_MOESM1_ESM.pdf]

SUPPLEMENTARY FIGURES AND TABLES

Delicate balance among thermal stability, binding affinity, and conformational space explored by single-domain V_HH antibodies

Emina Ikeuchi[1,2,†], Daisuke Kuroda[1,3,4,†], Makoto Nakakido[1,4], Akikazu Murakami[5], and Kouhei Tsumoto[1,3,4,6,*]

[1] Department of Bioengineering, School of Engineering, The University of Tokyo, Tokyo 108-8639, Japan
[2] Panasonic Corporation Technology Division, Kyoto 619-0237, Japan
[3] Medical Device Development and Regulation Research Center, School of Engineering, The University of Tokyo, Tokyo 108-8639, Japan
[4] Department of Chemistry and Biotechnology, School of Engineering, The University of Tokyo, Tokyo, Japan
[5] Department of Parasitology and Immunopathoetiology, Graduate School of Medicine University of the Ryukyus, Okinawa 903-0215, Japan
[6] Laboratory of Medical Proteomics, The Institute of Medical Science, The University of Tokyo, Tokyo 108-8639, Japan

[†]These authors contributed equally to this work
*Correspondence should be addressed: KT (tsumoto@bioeng.t.u-tokyo.ac.jp)

Figure S1. SPR response profiles of the antibodies.

Figure S2. Binding curve from SPR analysis of the G78A mutant.

Figure S3. A cavity observed inside the predicted structure of Z18.

Figure S4. RMSD of Cα-atoms of entire antibody structures.

Figure S5. RMSD of Cα-atoms of each CDRs.

Figure S6. Fraction of native contacts.

Table S1. Structural templates used in antibody modeling.

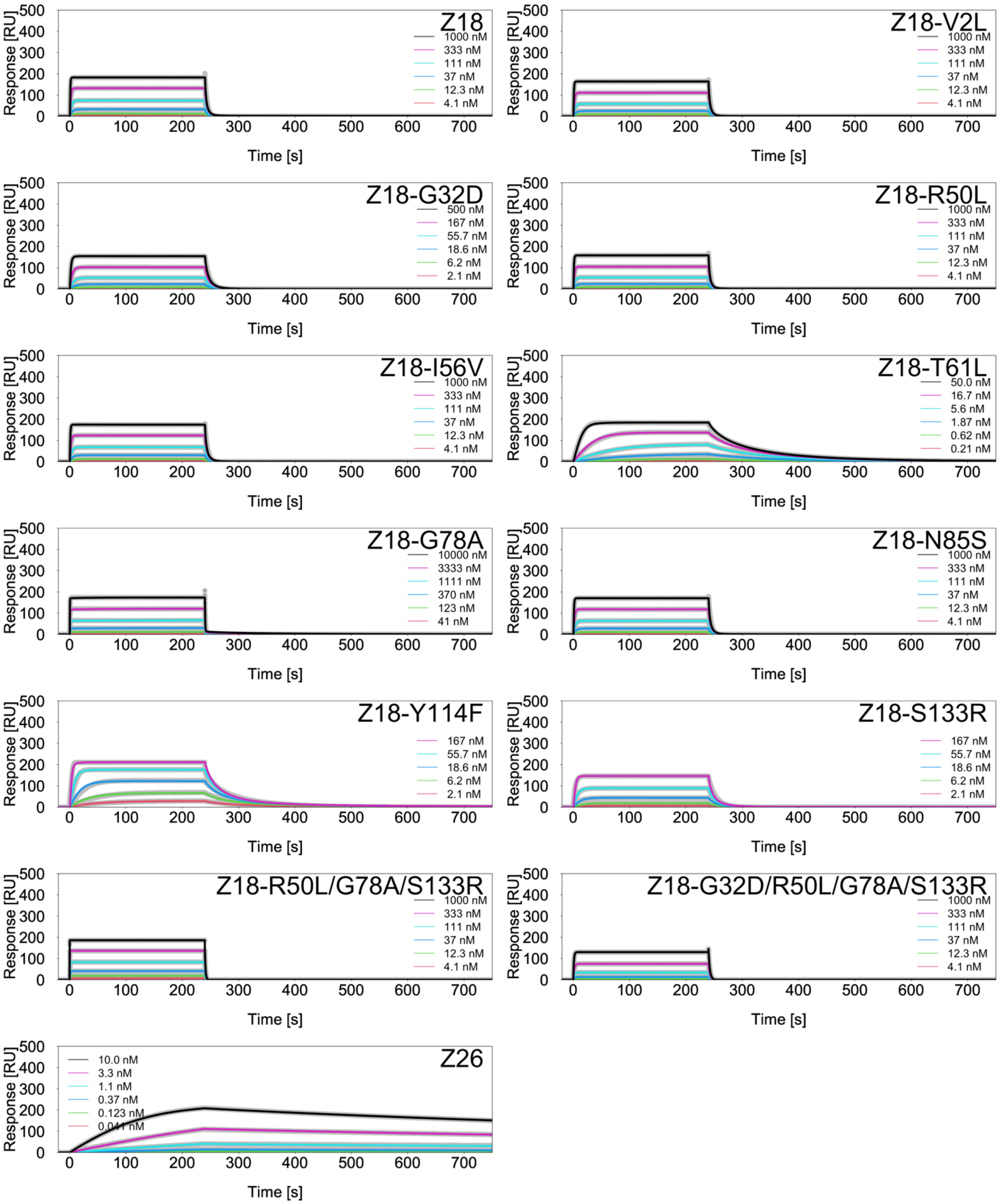

Figure S1. SPR response profiles (raw) of the antibodies. Fitting curves are shown in grey lines.

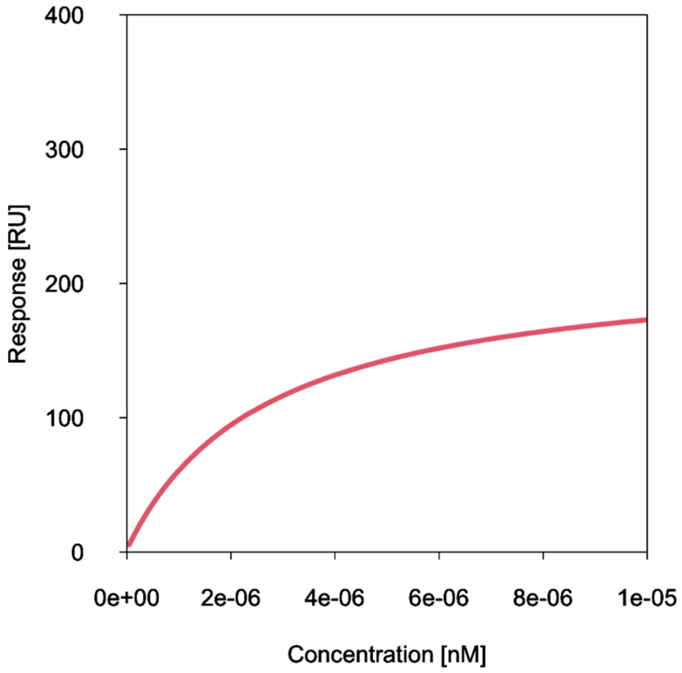

Figure S2. Binding curve from SPR analysis of the G78A mutant. Due to the low affinity (K_D = 7.11×10^{-6} M), the affinity was determined by steady state analysis.

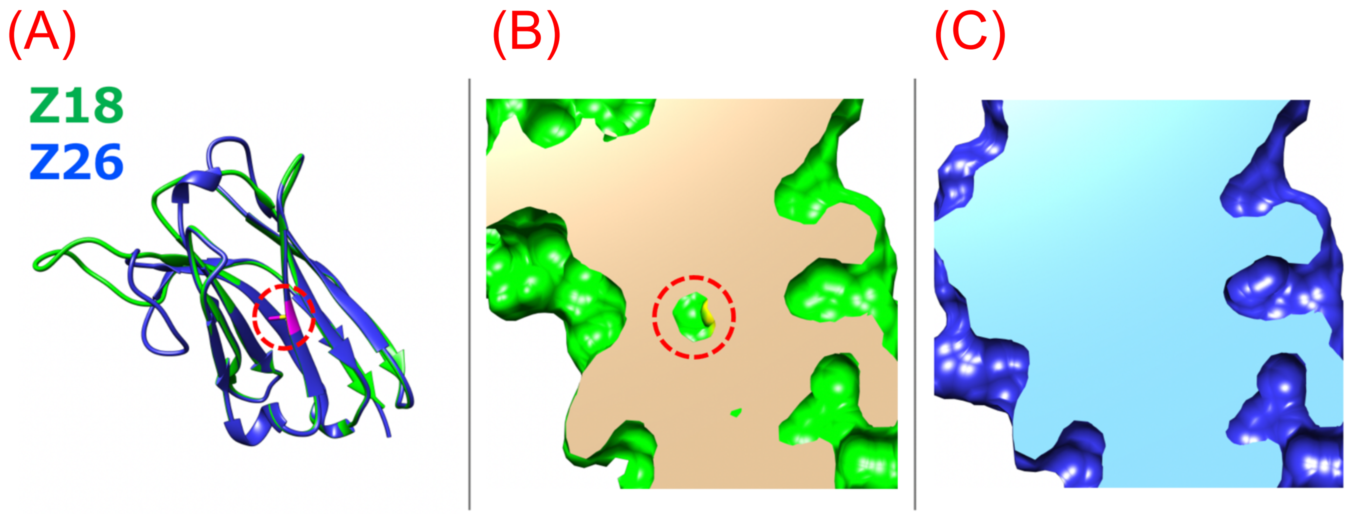

Figure S3. A cavity observed inside the predicted structure of Z18. (A) The superposed structures of Z18 (green) and Z26 (blue). The sliced models of (B) Z18 and (C) Z26. The cavity is highlighted by a red circle in (A) and (B).

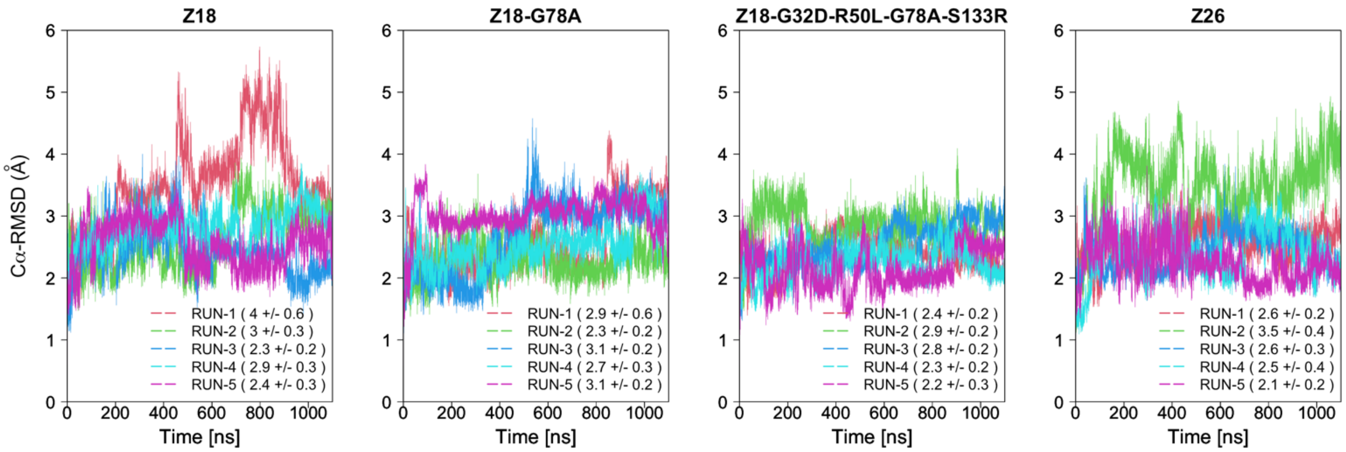

Figure S4. RMSD of Cα–atoms of entire antibody structures. The average and standard deviation are calculated using the last 500 ns trajectories and are provided in the parenthesis in each simulation.

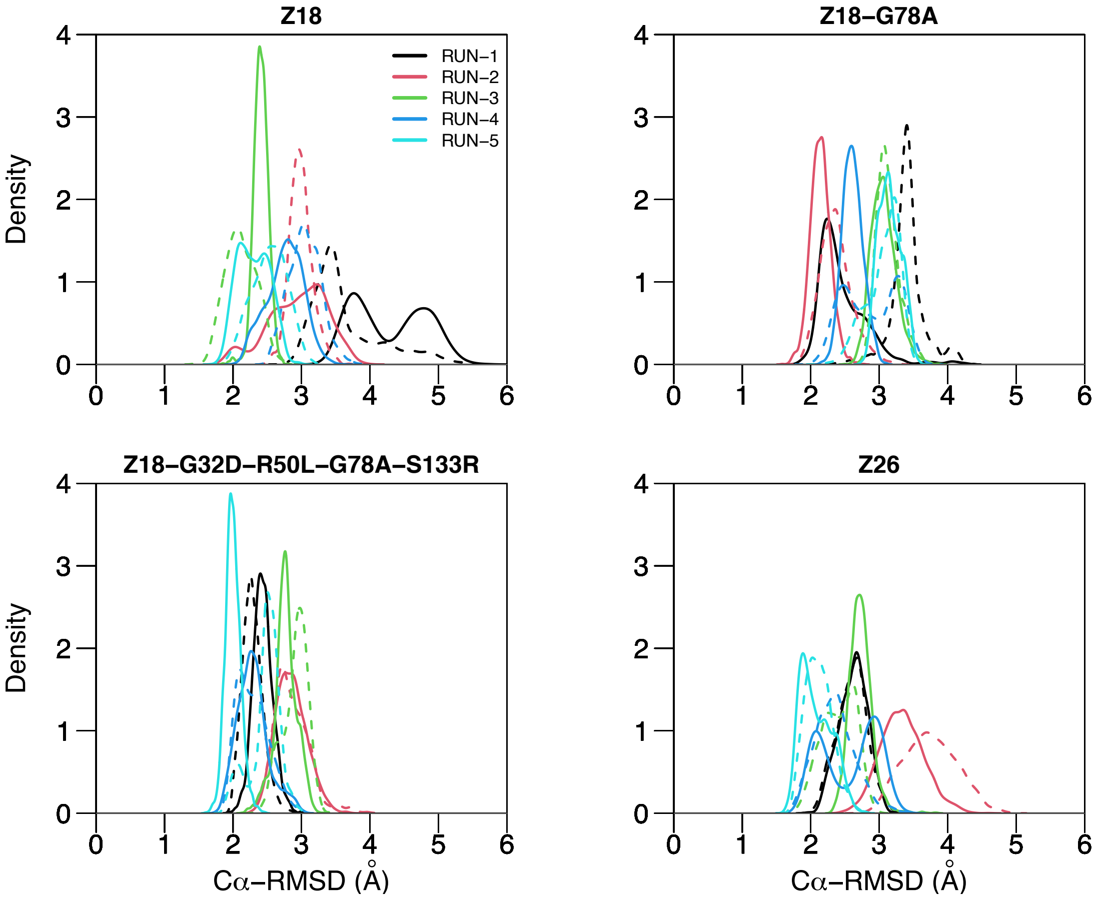

Figure S5. Substantial overlaps between the first half (straight line) and last half (dotted line) of each trajectory.

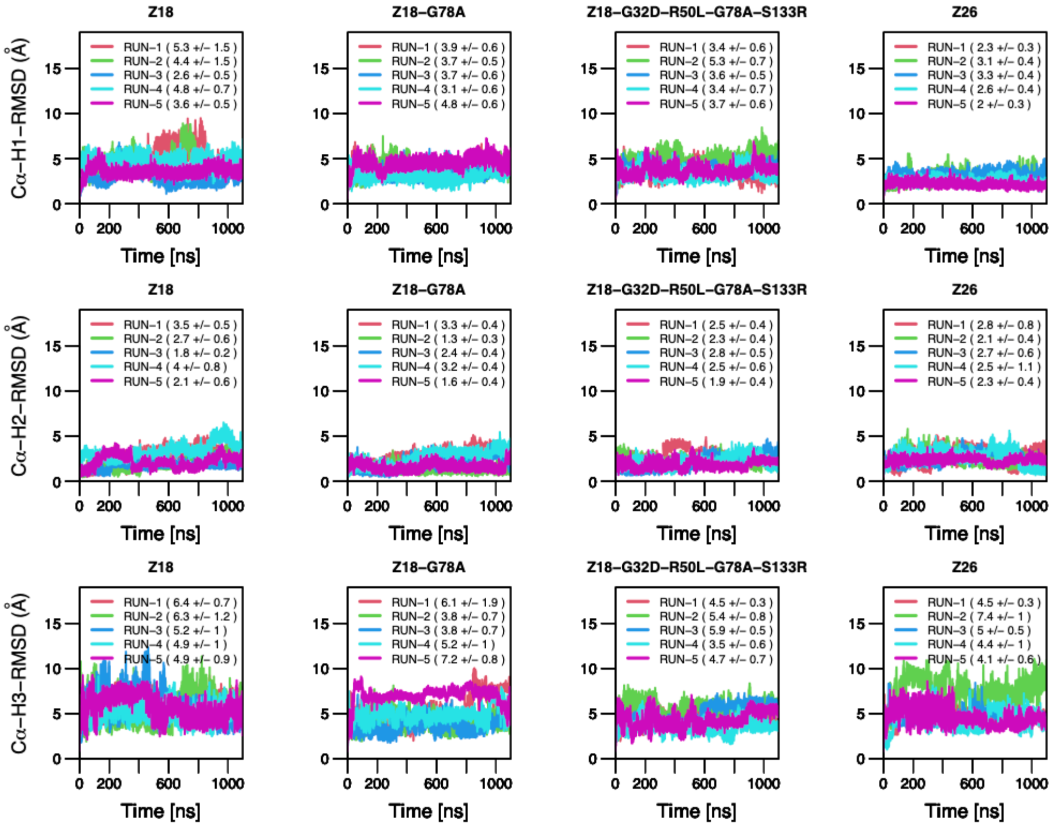

Figure S6. RMSD of Cα–atoms of each CDRs. The average and standard deviation are calculated using the last 500 ns trajectories and are provided in the parenthesis in each simulation.

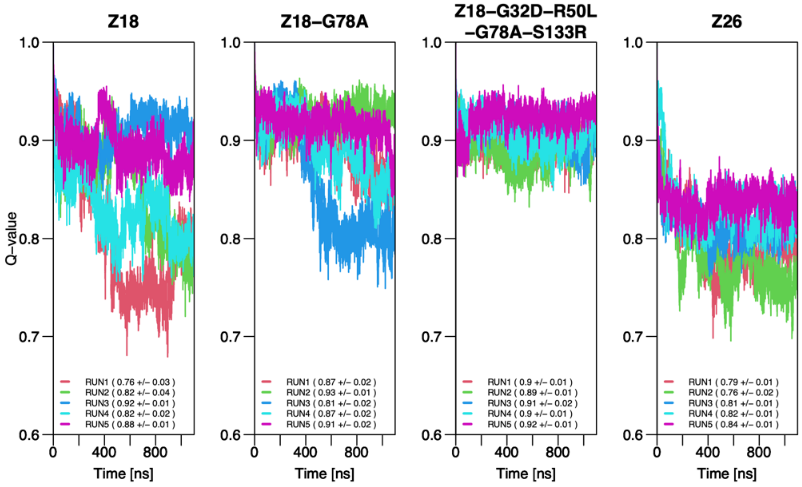

Figure S7. Fraction of native contacts. The average and standard deviation are calculated using the last 500 ns trajectories and are provided in the parenthesis in each simulation.

Table S1. Structural templates used in antibody modeling. The PDB ID is shown for each component.

	Z18	Z26
CDR-H1	4krn	4qlr
CDR-H2	4qo1	4qo1
CDR-H3	5en2	2vyr
FRH	4o9h	1dlf

Table S2. Convergence of each trajectory. The average and standard deviation are calculated using the last 500 ns trajectories.

	Z18	Z18-G78A	Z18-G32D-R50L-G78A-S133R	Z26
RUN-1	4.0 ± 0.6[1]	2.9 ± 0.6	2.4 ± 0.2	2.6 ± 0.2
RUN-2	3.0 ± 0.3	2.3 ± 0.2	2.9 ± 0.2	3.5 ± 0.4
RUN-3	2.3 ± 0.2	3.1 ± 0.2	2.8 ± 0.2	2.6 ± 0.3
RUN-4	2.9 ± 0.3	2.7 ± 0.3	2.3 ± 0.2	2.5 ± 0.4
RUN-5	2.4 ± 0.3	3.1 ± 0.2	2.2 ± 0.3	2.1 ± 0.2

[1] Since the RMSD values deviated from those of the other 4 simulations, we have excluded the trajectory from subsequent analyses.
